# Effect of pegbovigrastim on clinical mastitis and uterine disease during a full lactation in grazing dairy cows

**DOI:** 10.1371/journal.pone.0252418

**Published:** 2021-05-27

**Authors:** Joaquín Barca, Ana Meikle, Mette Bouman, Giovanni Gnemmi, Rodrigo Ruiz, Ynte H. Schukken

**Affiliations:** 1 Department of Dairy Science and Technology, Veterinary Faculty, Montevideo, Uruguay; 2 Department of Animal Sciences, Wageningen University, Wageningen, the Netherlands; 3 Animal Endocrine and Metabolism Laboratory, Veterinary Faculty, Montevideo, Uruguay; 4 Veterinary Practitioner, Colonia, Uruguay; 5 Bovinevet Internacional Bovine Ultrasound Services & Herd Management, Huesca, Spain; 6 Department of Animal Reproduction Veterinary Faculty, Catholic University of Valencia, Valencia, Spain; 7 Diamond V, Corregidora, Querétaro, México; 8 GD Animal Health, Deventer, the Netherlands; National Veterinary School of Toulouse, FRANCE

## Abstract

In this randomized controlled trial on four commercial grazing dairy farms, we investigated whether pegbovigrastim (PEG) treatment affects clinical mastitis (CM) and uterine disease (i.e. retained placenta (RP), metritis and endometritis) occurrence during a full lactation. The association of prepartum body condition score and prepartum non-esterified fatty acid (NEFA) concentration with disease occurrence was also evaluated. Holstein cows were randomly assigned to one of two treatments: first PEG dose approximately 7 d before the expected calving date and a second dose within 24 h after calving (PEG) compared to untreated controls (Control). In total, 2,153 animals were included in the study: 733 primiparous cows (Control = 391, PEG = 342) and 1420 multiparous cows (Control = 723, PEG = 697). Treatment effects were evaluated with generalized linear mixed models and Cox’s proportional hazard models. Treatment with PEG reduced the occurrence of a first case of CM during the first 30 days in milk (DIM) by 24.6% and reduced the hazard of a first case and the rate of total cases of CM during the full lactation. All PEG treatment effects were independent of parity. Prepartum body condition score interacted with PEG treatment: in over-conditioned cows, PEG reduced the occurrence of a first case of CM during the first 30 DIM by 49.5%. The hazard analysis of a first case of CM during the full lactation suggested that the preventive effect of PEG disappeared with increasing DIM. Treatment with PEG did not affect the occurrence of RP or metritis. Pegbovigrastim treated cows with metritis subsequently showed a reduced occurrence of endometritis compared to control cows with metritis. Pegbovigrastim reduces the occurrence of CM particularly in cows at risk of elevated lipid mobilization, and PEG ameliorates the uterine healing process in cows that experienced metritis.

## Introduction

The high prevalence of metabolic and infectious diseases that dairy cows experience during the first month of lactation is a main concern to the dairy industry. The risk for disease in early lactation has been associated, among other causes, with the negative energy balance (NEB) that takes place during the transition period [1]. Indeed, increased concentrations of non-esterified fatty acids (NEFA), related to the NEB, have been linked to immunosuppression and increased risk of mastitis, retained placenta (RP) and metritis [2–6]. The rate and extent of energy mobilization from fat tissues has been linked to an increased risk of periparturient metabolic and clinical disorders during the transition period [2, 7]. Roche et al. [7] reported that over-conditioned cows (BCS > 3.5)—that mobilize more fat reserves than cows with a moderate BCS (3–3.5)—had more mastitis, and suggested that high BCS is related to an impaired energy and lipid metabolism that may affect the immune response in these cows. Although the relationship between thin cows and the risk of periparturient metabolic disorders is less consistent [7], cows with a low BCS (BCS < 3) had more mastitis relative to cows with a moderate BCS, and low BCS has also been reported as a risk factor for uterine disease [7, 8].

One of the latest developments in preventive tools is the use of a long-acting analogue of bovine granulocyte colony-stimulating factor (PEG, Pegbovigrastim, marketed as Imrestor® by Elanco Animal Health, Greenfield, IN). Pegbovigrastim increases circulating white blood cell (WBC) and neutrophil counts and myeloperoxidase exocytosis [9, 10]. Recently, we found that prepartum NEFA (Pre-NEFA) concentrations were associated with reduced neutrophil counts, and that PEG treatment reversed this negative association [11]. It has been reported that treatment with PEG reduced the occurrence of clinical mastitis (CM) during the first 30 days in milk (DIM). However, the percentage reduction in CM varied substantially, ranging from 23% to 50% [12, 13]. A more recent study found no effect on the occurrence of CM during the first 30 DIM [14]. Evidence for the use of PEG to reduce metritis occurrence has also been inconsistent: Ruiz et al. [13] reported an increase, Zinicola et al. [14] no effect and Freick et al. [15] a decrease.

Parity is a major factor that impacts the metabolic adaptation to lactation, as in primiparous cows the requirements for growth limit nutrient partitioning into milk. While NEFA, as a proxy of lipid mobilization, were lower in primiparous vs multiparous cows in confined systems [16], a more pronounced NEB was observed in primiparous cows under grazing conditions [17, 18]. Under confined feeding conditions, the quantity and quality of nutrients for dairy cows can be controlled; however, when pasture is the main component of the diet, nutrient intake estimation is more complex [19]. Typically, primiparous cows have a higher risk of early lactation CM and metritis, although multiparous cows have more milk fever and CM throughout the full lactation [20–22]. To our knowledge, the impact of PEG on disease occurrence by parity in grazing herds has not yet been reported.

The hypothesis for this study was that PEG treatment reduce disease occurrence in grazing dairy cows and that the response to treatment would be associated with parity, prepartum BCS (Pre-BCS) and/or Pre-NEFA. Thus, we investigated whether PEG treatment affected disease occurrence during a full lactation in primiparous and multiparous grazing dairy cows, and evaluated the association of Pre-BCS and Pre-NEFA concentration with disease occurrence.

## Materials and methods

The experimental protocol (CEUAFVET-PI-162) was evaluated and approved by the Honorary Committee for Animal Experimentation in Uruguay (CHEA), University of Uruguay.

### Study design

Holstein primiparous and multiparous cows (n = 2,333) from 4 commercial grazing dairy farms in 3 different regions of Uruguay (San José, Florida, Rio Negro) were included in this prospective randomized controlled trial. Farms were selected for convenience; inclusion was based on excellent record keeping, a willingness to participate in the study and the ability to implement the research protocol. Farms 1, 3 and 4 had a seasonal calving system, with calving concentrated in autumn. These herds had a milking herd size of approximately 1,000, 850 and 600 cows, respectively. Farm 2 had a continuous year-round calving system and a milking herd size of approximately 600 cows. Throughout this manuscript, primiparous animals are cows that were enrolled in the study shortly before their first calving. Multiparous animals are cows that were enrolled shortly before their second or higher calving. The follow-up period for all cows with regard to clinical events was a full lactation (305 DIM). Recorded reasons for exit from the study were: dry-off, culling, and death. In animals for which none of these exit reasons were recorded, 305 DIM was considered the (censored) end point of the study.

All farms used blanket antibiotic dry cow therapy, antibiotics to treat CM cases and antiseptic post-milking teat dips. The cows from all 4 farms were moved to outdoor close-up paddocks around 3 weeks before the expected calving date. Calving occurred in the same area or in a subdivision of the same paddock under the same conditions. Calving for cows enrolled in the study occurred between February 13th and September 30th of 2018. After calving, cows were kept on pasture at least one of the periods between the two daily milkings and at least 40% of the dry matter intake came directly from the grazing sessions, supplemented with a partial mixed ration.

Three veterinary technicians were hired and trained as research assistants for this experiment. Technicians enrolled animals, administered experimental treatments, took blood samples, assessed BCS, diagnosed diseases or confirmed diagnoses made by the farm staff, and were responsible for keeping written records. Two farms had a full time veterinary technician per farm, while two farms shared one technician. On the latter two farms the technician was supported on a daily basis by the first author.

The time of enrollment in the study was between -10 to -7 days relative to the expected calving date (ECD). Animals that had fever (rectal temperature > 39.5°C) or any other clinical health disorder at the time of enrollment were excluded from the study. Animals that met the inclusion criteria were assigned to one of two treatments based on their national ear tag number. The national ear tag numbers are available from computer records but are independent of the large and easily visible ear tag number (cow ID) that is used for farm management. Animals with an even national ear tag number (randomization was carried out by a single flip of a coin before the start of the study) were injected with PEG (Imrestor®, Elanco Animal Health, Greenfield, IN) according to the product label (PEG). Briefly, periparturient dairy cows received a subcutaneous injection of 15 mg of PEG approximately 7 d before their ECD and within 24 h of calving. Animals with an odd national ear tag number remained as untreated controls (Control). No placebo was used, as treatment allocation based on the national ear tag number provided sufficient blinding. Close-up paddocks were observed twice a week. Cows that were between -10 to -7 days relative to the expected calving date or were exhibiting clinical signs of calving such as swelling of the vulva and filling of the udder were clinically examined. Cows that met any of the exclusion criteria (i.e. fever or any other clinical disease) were excluded, while all other cows were included in the study. Only cows that received the two doses were included in the analyses. Blood samples were obtained from Control and PEG cows at -10 to -7 days relative to the expected calving date and within 24 h after calving (this second sample was taken for other purposes beyond the objectives of this study). The included animals therefore represent the ‘per protocol’ inclusion rule [23]. Farm personnel (including milkers) and laboratory personnel were blinded to treatment status. Research technicians applied treatments and would therefore be aware of the treatment status. However, all animal observations, samplings and disease diagnoses were based on the visible on-farm cow ID number that was unrelated to the national ear tag number that was used for randomization.

Our objective was to enroll 2,400 animals. This number was based on a power calculation assuming a CM incidence during the first 30 DIM of 15% and 11.25% for Control and PEG treated animals respectively. Canning et al. [10] reported a reduction in the incidence of CM of 35%, and a field trial conducted for authorization of use of PEG in the European Union [24] showed a reduction in the incidence of CM of 26%. We assumed an efficacy of 25% to be on the safe side. Power calculations (α = 0.05, β = 0.20) then showed that a sample size of 1,200 cows per treatment group was needed.

At day -10 to -7 from the expected calving date (enrollment), Pre-BCS was assessed and recorded by the veterinary technicians. At the same time, blood samples were collected from the coccygeal vessels (8.5-mL clot accelerator tubes, Becton Dickson, Franklin Lakes, NJ). Immediately, blood samples were centrifuged at 3,000 x g for 20 min and serum was stored frozen (-20°C) until further analysis for NEFA concentration.

### Clinical diagnoses and definitions

Each veterinary technician was trained prior to the start of the study to assess BCS (1 to 5 score [25]) and to diagnose clinical events including CM, RP, metritis, clinical endometritis, metabolic disorders (milk fever and ketosis) and lameness (see definitions below). All farm personnel were also trained in the recognition of these disorders and all diagnoses were ultimately confirmed by the trial technicians. At two post-partum visits, the first at 5 to 8 and the second at 27 to 30 d of lactation, all cows were carefully examined by the veterinary technician to diagnose metritis and endometritis respectively. The diagnosis was made by vaginal examination using a clean palpation glove. If metritis and clinical endometritis were diagnosed by the farm personnel at a different time point, this was also recorded and included into the disease categories described hereunder.

Clinical mastitis was diagnosed by trained farm personnel during forestripping prior to each milking. Clinical mastitis was scored according to Pinzón-Sánchez and Ruegg [26] as mild (abnormal milk without other symptoms), moderate (abnormal milk and local symptoms in the udder), or severe (abnormal milk, local symptoms and also signs of systemic illness). Farm staff were instructed to record all treated cases of CM, irrespective of the time since a previous case; a single event per treatment protocol was recorded, irrespective of the number of affected quarters. All CM cases, irrespective of severity, were reported as CM. Retained placenta was defined as fetal membranes (placenta) still visibly hanging from the cow´s vulva 24 h after calving [13]. Puerperal metritis was diagnosed if an animal showed a fetid watery red-brown uterine discharge, associated with signs of systemic illness (such as decreased milk yield, dullness or other signs of toxaemia) and fever (rectal temperature > 39.5°C or > 40.5°C during summer and when ambient temperature was higher than 30°C [27]), within 21 days post-partum. Clinical metritis was defined as a purulent uterine discharge detectable in the vagina in the first 21 days after calving without systemic illness. Puerperal metritis and clinical metritis were combined and reported as metritis. Clinical endometritis was defined as the presence of purulent uterine discharge visible in the vagina 21 days or more post-partum, or mucopurulent discharge visible in the vagina more than 26 days post-partum. Manual vaginal examinations were performed using clean palpation gloves. All these uterine diseases were defined according to Sheldon et al. [28]. Milk fever was defined as either a standing animal showing mild ataxia, excitability, muscle tremors and reduced ruminal motility or a recumbent cow [29, 30]. Clinical ketosis was defined according to Kelton et al. [29] as an animal with decreased appetite in the absence of another concurrent disease. Lameness was defined as animals with clinical signs of abnormal locomotion [31].

### Non-esterified fatty acids determination

Non-esterified fatty acid concentrations were determined at the Animal Endocrine and Metabolism Laboratory, Veterinary Faculty, Montevideo, Uruguay. Colorimetric assays were performed on an A25 autoanalyzer (© Biosystems S.A., Barcelona, Spain) using commercial kits: Wako NEFA-HR (2), Wako Pure Chemical Industries Ltd., Osaka, Japan. The inter-assay coefficient of variation (CV) for commercial quality controls was less than 10%.

### Statistical analysis

Data were analysed using SAS software (SAS Institute Inc. 2018. SAS^®^ University Edition, Cary, North Carolina: SAS Institute Inc.).

Descriptive statistics were performed using the t-test procedure (PROC TTEST) and chi-squared tests for continuous and categorical variables respectively. Categorical variables included last test day SCC of the previous lactation (low ≤ 200,000 cell/mL, high > 200,000 cell/mL), recorded as SCC at dry off, and occurrence of CM cases in the previous lactation (yes/no). The frequency procedure (PROC FREQ) was used to group cows by Pre-BCS (under: < 3; acceptable: 3 to 3.5, and over: > 3.5; [7]) and Pre-NEFA categories (low ≤ 0.5 m*M*, high > 0.5 m*M*) by treatment group.

Logistic regressions to analyze occurrence of a first case of CM during the first 30 DIM and clinical uterine diseases (i.e. RP, metritis, and endometritis) were performed using the generalized linear mixed model procedure (PROC GLIMMIX). Fixed effects in the model included as class variables were: treatment (Control/PEG), parity (primiparous/multiparous), Pre-BCS (under: < 3; acceptable: 3 to 3.5 and over: > 3.5), Pre-NEFA category (low ≤ 0.5 m*M*, high > 0.5 m*M*) and calving month (1 to 6: February/March, April, May, June, July and August/September, respectively). Two-way interactions with treatment were checked for significance. Farm, as a class variable, was included as a random effect. In the CM model, SCC at dry off was included as a class variable (low ≤ 200,000 cell/mL, high > 200,000 cell/mL, primiparous cows were coded as low) and the interaction with treatment was checked. In the metritis model, RP occurrence was included as a class variable. In the endometritis model, both RP and metritis were included as class variables. All class variables were coded in the class statement in the models.

The initial statistical model looked like:

Logit (disease) = intercept + parity + treatment + calving month + Pre-BCS + Pre-NEFA + SCC at dry off (for CM) + RP (for metritis and endometritis) + metritis (for endometritis) + interactions + farm (random) + error.

After an initial full model lay-out, a backward stepwise selection process was performed.

Solutions for fixed effects are presented as comparisons with the reference groups specified in each table of results, and where relevant, estimated least squares means are presented. The overall treatment effect (type III tests of fixed effects) and estimated least squares means differences between Control and PEG cows are also presented.

A Cox’s proportional hazard model was performed (PROC PHREG) to analyze the hazard of a first case of CM during a full lactation (305 DIM, right censored). This model included the same variables as the logistic regression described above. Additionally, a variable called Days-block (DIM blocked in 60 day intervals) was included, and the interaction with treatment was checked to assess a possible differential effect of treatment during the course of the lactation. A forward stepwise selection process was performed.

A Poisson regression model was performed to evaluate the effect of PEG on the total number of CM cases during the full lactation (PROC GLIMMIX). All cases of CM at cow level were included in the analyses, including those occurring within 14 days from a previous case as these early repeat cases did require antibiotic treatment and resulted in milk withhold. In the Poisson model, the number of CM cases during the full lactation was the outcome variable and the log number of days at risk was included as an offset. This model included the same variables as described above, with farm as a random effect. A forward stepwise selection process was performed.

In all analyses, treatment and parity were always forced into the models. All other variables or their two-way interaction with treatment with a *P* ≤ 0.10 remained in the model during the variable selection process. Exceptionally, variables or their interaction term with treatment with a *P* ≤ 0.15 remained in the model, but only when removal of the variable resulted in a considerable (> 20%) change in the estimate of treatment. Such variables would be considered potential confounders. Statistical tendency and significance were set at *P* ≤ 0.10 and *P* ≤ 0.05 respectively.

## Results

### Study population

A total of 2,333 cows were initially enrolled, of which 116 did not meet the per protocol inclusion rule: 89 cows (Control = 30, PEG = 34 and no treatment data = 25) never calved and the farm veterinarian eventually diagnosed these cows as not pregnant; 5 cows (Control = 3 and PEG = 2) were enrolled twice and the second enrollment was removed; 5 cows (Control = 2, PEG = 2 and no treatment data = 1) did not have the date of inclusion recorded. In addition, PEG = 13 cows were injected at the time of inclusion but not injected at calving, and Control = 4 cows were erroneously injected at calving. Finally, 64 cows (Control = 30, PEG = 34) had no Pre-NEFA determination. Thus, 2,153 cows were considered in the final analyses: 733 primiparous cows (Control = 391, PEG 342) and 1,420 multiparous cows (Control: 723, PEG = 697).

### Balance between treatment groups

No differences between treatment groups were found for actual lactation number, with average values of 2.4 ± 1.5 and 2.5 ± 1.5 for Control and PEG cows respectively (*P* = 0.37). Table 1 presents descriptive data of the previous lactation for the enrolled multiparous cows by treatment group. It includes lactation number at enrollment, previous milk production, DIM at dry off, daily milk production, proportion of cows with high SCC at dry off, and proportion of cows with one or more clinical mastitis cases in previous lactation. No differences between treatment groups were found.

**Table 1:** Descriptive data from the lactation before enrollment in the study for multiparous cows.

	Treatment (Mean ± SD)		
Item	Control	PEG	P-value
Lactation number at enrollment	2.2 ± 1.4	2.2 ± 1.3	0.83
Previous lactation milk (kg)	7,636 ± 2,273	7,543 ± 2,197	0.43
Days in milk at dry-off	363 ± 102	354 ± 92	0.14
Daily milk (kg/day) previous lactation	21.4 ± 5.2	21.7 ± 5.4	0.31
Occurrence of CM cases (%, n)*	38 (276)	39 (269)	0.87
SCC at dry off (%, n)**	54 (388)	57 (398)	0.19

Control = 723; PEG = 697.

*32 and 34 Control and PEG cows respectively have no previous data of CM.

**proportion of cows with high (> 200,000 cell/mL) SCC at the last test day of previous lactation.

**Fig 1:**
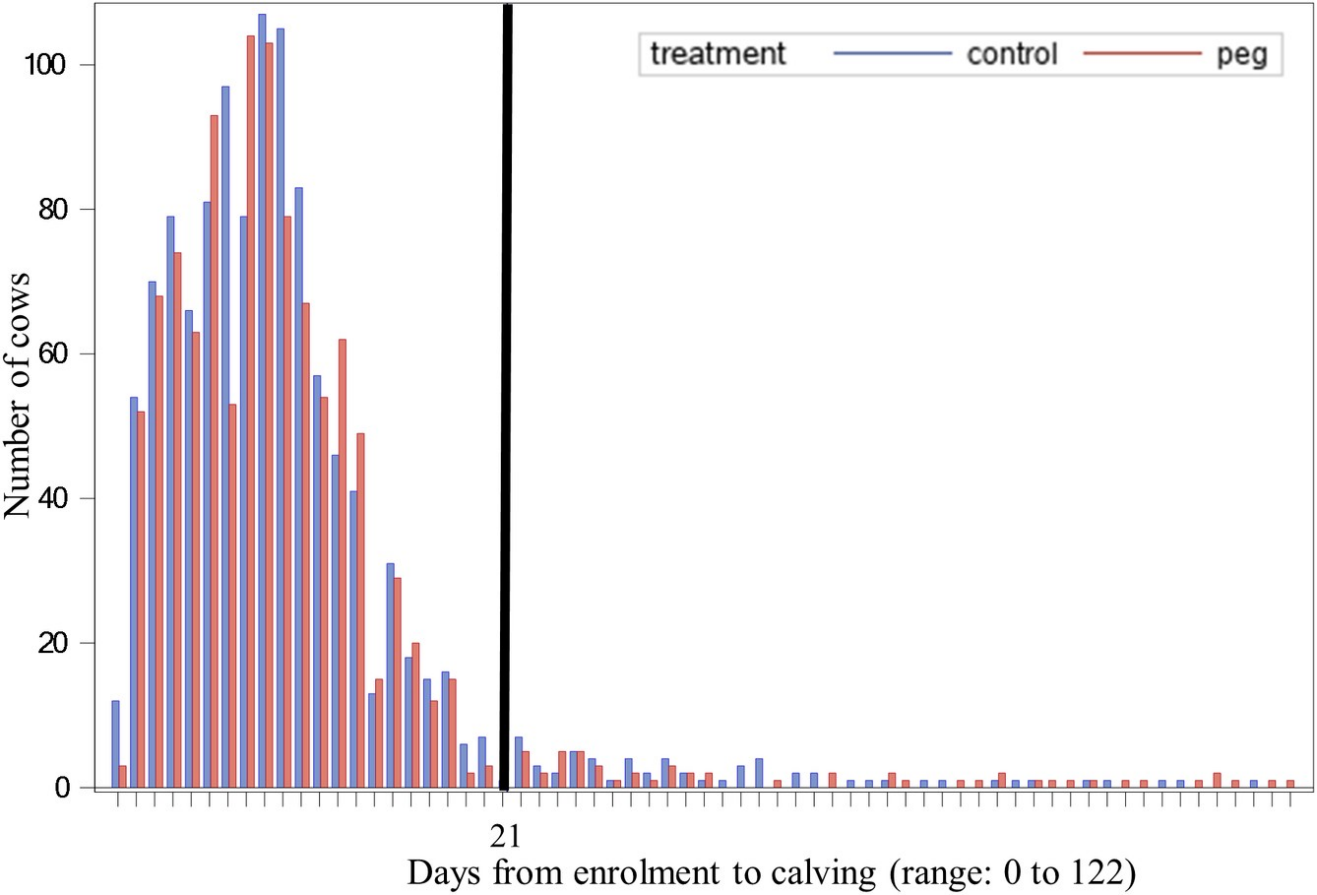
Number of cows by interval from enrollment to calving (day 0). Ninety six percent of the animals were enrolled within 21 days (black line) before calving. Control = 1,114; PEG = 1,039. Note: some values in the range have frequencies of 0 so are not represented in the x-axis.

Fig 1 shows the number of cows by days between enrollment and calving for the two treatment groups. The mean values and SD were 9 ± 8 and 9 ± 10 days for Control and PEG cows respectively (*P* = 0.42). Primiparous Control and PEG cows were enrolled at 10 ± 10 and 10 ± 12 days before calving (*P* = 0.90) and multiparous Control and PEG cows at 9 ± 7 and 9 ± 9 days before calving (*P* = 0.26). Forty eight percent of the animals were enrolled within one week before calving, 41% between one and two weeks before calving and 7% between two and three weeks before calving, so that 96% of the cows were enrolled within 21 days before calving.

No differences between treatment groups were found for BCS, with average scores of 3.4 ± 0.4 and 3.4 ± 0.4 for Control and PEG cows respectively (*P* = 0.29). The number of cows in each Pre-BCS category was: Under: Control = 107; PEG = 99; Acceptable: Control = 702; PEG = 706 and Over: Control = 305; PEG = 234. No differences between treatment groups were found for Pre-NEFA concentration (0.50 ± 0.39 and 0.52 ± 0.42 m*M* for Control and PEG cows respectively, *P* = 0.47). The number of cows in each Pre-NEFA category was: Low (≤ 0.5 m*M*): Control = 667; PEG = 621: High (> 0.5 m*M*): Control = 447 PEG = 418.

Differences between parity groups were found for Pre-BCS, with scores of 3.5 ± 0.4 and 3.3 ± 0.4 for primiparous and multiparous cows respectively (*P* < 0.001) and for Pre-NEFA concentrations: 0.61 ± 0.42 and 0.46 ± 0.38 m*M* for primiparous and multiparous cows respectively (*P* < 0.001).

### Disease occurrence

In total, 31 cows (Control = 17, PEG = 14) recorded milk fever and 6 cows (Control = 6, PEG = 0) clinical ketosis. A total of 223 (Control = 120, PEG = 103) cows recorded lameness. However only 45 (Control = 22, PEG = 23) had a case during the first 30 d of lactation. These clinical diseases were not further analyzed in regression analyses.

### Effect of pegbovigrastim on clinical mastitis in primiparous and multiparous cows

A total of 2,005 (Control = 1,077, PEG = 928) cases of CM were recorded.

**Fig 2:**
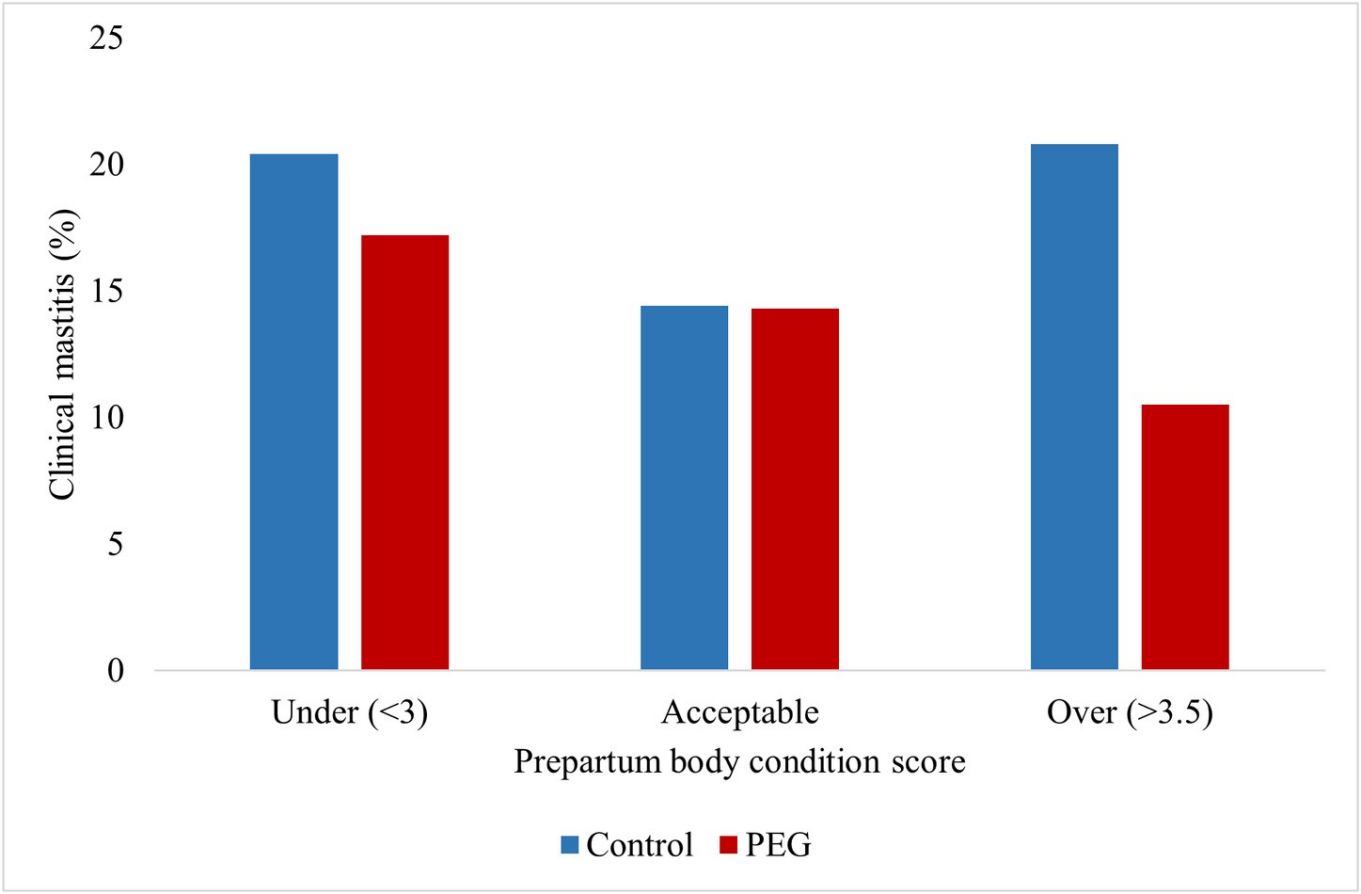
First case of clinical mastitis during the first 30 DIM by prepartum body condition score. Under-conditioned (< 3; Control = 107; PEG = 99); Acceptable-conditioned (3–3.5; Control = 702; PEG = 706); Over-conditioned (>3.5; Control = 305; PEG = 234). In over-conditioned cows, occurrence of CM was lower in PEG treated cows (*P* < 0.05).

Regression analysis results for the occurrence of a first case of CM during the first 30 DIM are presented in Table 2. Treatment with PEG reduced the occurrence of a first case of CM during the first 30 DIM (*P* = 0.002). Occurrence of a first case of CM during the first 30 DIM was not associated with parity (*P* = 0.50) and no treatment by parity interaction was detected. Cows with an acceptable BCS had a lower occurrence of a first case of CM during the first 30 DIM compared with over-conditioned cows (*P* = 0.02). Prepartum BCS interacted with treatment: the preventive effect of PEG was not observed in cows with an acceptable Pre-BCS (*P* = 0.008). Fig 2 presents least squares means differences of occurrence of a first case of CM during the first 30 DIM by Pre-BCS in control and PEG cows. In over-conditioned cows, PEG reduced the occurrence of a first case of CM during the first 30 DIM by 49.5% (Control = 20.8%, PEG = 10.5%; *P* = 0.02), while no significant reduction was detected in under-conditioned cows (Control = 20.4%, PEG = 17.2%; *P* = 0.55) and in cows with an acceptable BCS (Control = 14.4%, PEG = 14.3%; *P* = 0.96).

**Table 2:** Occurrence of a first case of clinical mastitis during the first 30 days in milk.

Effect	Estimate	SE	P–value
Intercept	-1.36	0.30	0.02
Treatment	-0.99	0.32	0.002
Parity, 1 vs >1	-0.11	0.17	0.50
SCC at dry off	0.41	0.15	0.006
Prepartum BCS			
Under*	-0.03	0.30	0.92
Acceptable**	-0.44	0.20	0.02
Pre-NEFA	-0.25	0.18	0.17
Prepartum BCS x Treatment			
Under*	0.60	0.44	0.17
Acceptable**	0.80	0.30	0.008
Pre-NEFA x Treatment	-0.36	0.25	0.15

Reference groups: Control group, prepartum BCS: Over-conditioned cows (BCS > 3.5), Pre-NEFA: high (> 0.5m*M*); SCC at dry off, 0 (< 200.000 cell/mL).

*Under-conditioned cows (BCS < 3)

**Acceptable BCS cows (3 to 3.5).

The Pre-NEFA by treatment interaction did not reach significance (*P* = 0.15); however, removing this variable changed the estimate of treatment considerably (from -0.99 -as shown in Table 2- to -0.70). When we analyzed least squares means differences, we found a differential effect of treatment with regard to Pre-NEFA. While no significant reduction due to PEG treatment was detected in low Pre-NEFA cows (Control = 16.5%, PEG = 14.4%; *P* = 0.39), treatment with PEG significantly reduced the occurrence of a first case of CM during the first 30 DIM (by 35.5%) in high Pre-NEFA cows (Control = 20.3%, PEG = 13.1%; *P* = 0.01).

When testing the overall treatment effect (type III test), we found that treatment with PEG reduced the occurrence of a first case of CM during the first 30 DIM by 24.6% (Control = 18.3%, PEG = 13.8%; *P* = 0.03).

**Fig 3:**
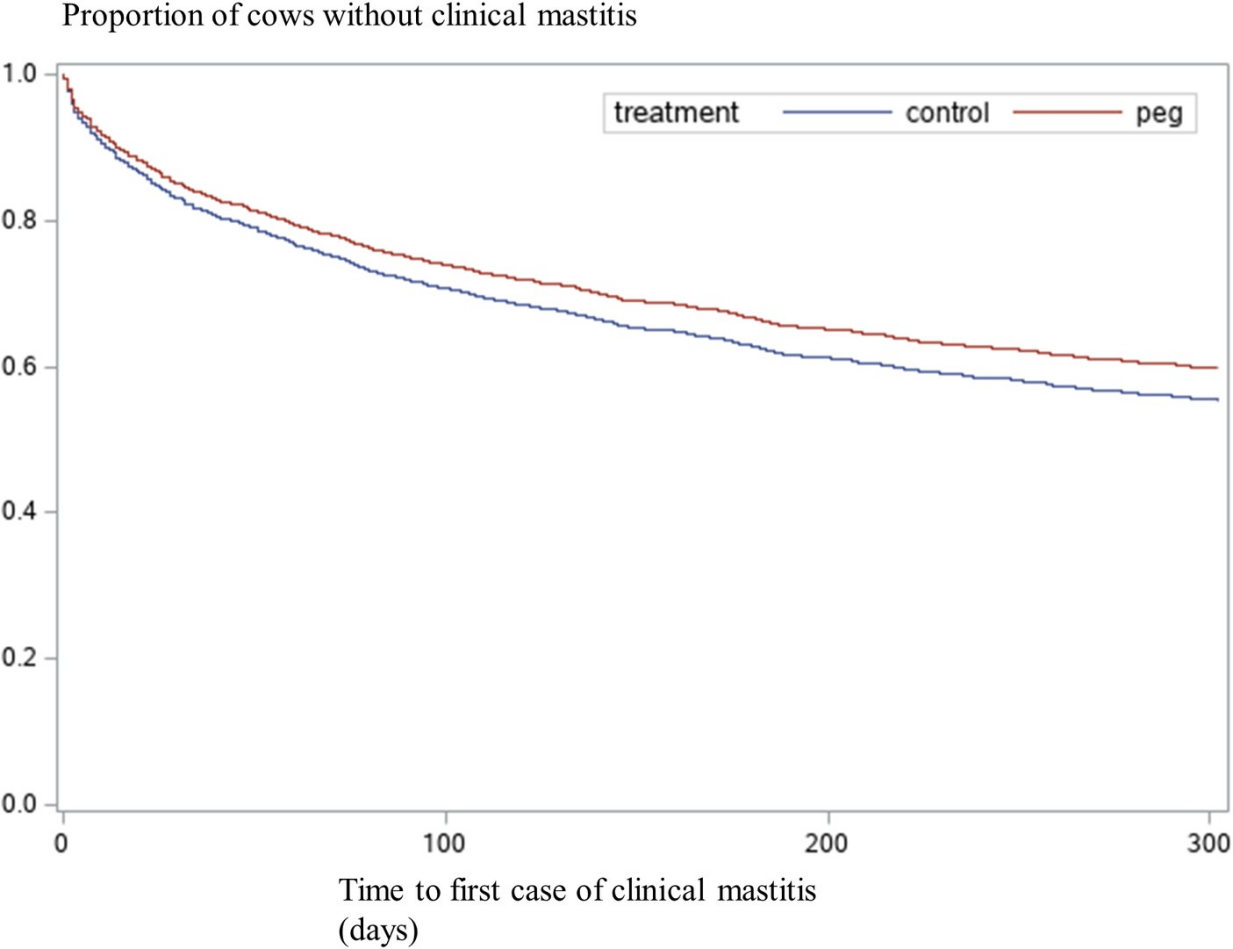
Survival curves of time to first case of clinical mastitis. Control cows = 1,114, PEG cows = 1,039. Treatment *P*–value = 0.008 (Table 3).

The hazard of a first case of CM during the full lactation is presented in Table 3. A significant treatment effect was detected: PEG cows had a 22% lower hazard of a first case of CM (HR = 0.78; *P* = 0.008). Parity was also significant: the hazard of a first case of CM was 24% lower in primiparous cows than in multiparous cows (HR = 0.76; *P* = 0.003). There was no treatment by parity interaction. The tendency towards significance of the interaction between treatment and Days-block (*P* = 0.09) suggested that the effect of treatment disappeared with increasing DIM (HR = 0.08 for each block of 60 DIM). Fig 3 shows the survival curves of time to first case of CM in control and PEG cows.

**Table 3:** Hazard of a first case of CM during a full lactation.

Effect	Estimate	SE	P—value	Hazard ratio
Treatment	-0.25	0.09	0.008	0.78
Parity, 1 vs >1	-0.28	0.09	0.003	0.76
SCC at dry off	0.37	0.08	< 0.001	1.45
Days-block* x Treatment	0.08	0.05	0.09	1.08

Reference group: Control group.

SCC at dry off 0 (< 200,000 cell/mL).

*Days-block were 60-day blocks for days of lactation.

The Poisson regression model (Table 4) showed a significant preventive effect of treatment on the rate of total cases of CM per cow-day at risk during the full lactation (*P* = 0.02). Parity was also significant, as primiparous cows had fewer total cases of CM (*P* < 0.001). No treatment by parity interaction was detected. Cows with an acceptable BCS had a lower rate of total cases of CM (*P* = 0.04).

**Table 4:** Rate of total cases of clinical mastitis per cow-day at risk during the full lactation.

Effect	Estimate	SE	P—value
Intercept	-5.77	0.13	<0.001
Treatment	-0.10	0.04	0.02
Parity, 1 vs >1	-0.34	0.06	<0.001
SCC at dry off	0.39	0.05	<0.001
Prepartum BCS			
Under*	-0.09	0.09	0.34
Acceptable**	-0.12	0.06	0.04
Calving month			< 0.001

Reference groups: Control group, prepartum BCS: Over-conditioned cows (BCS > 3.5), SCC at dry off 0 (< 200.000 cell/mL).

*Under-conditioned cows (BCS < 3)

** Acceptable BCS cows (3 to 3.5). Offset in the model was the natural log of the days-at-risk.

#### Effect of pegbovigrastim on clinical uterine diseases in primiparous and multiparous cows

The results of the regression analysis for clinical uterine disease are presented in Table 5.

**Table 5:** Regression analysis model results for uterine disease.

Uterine disease	Effect	Estimate	SE	P—value
Retained placenta	Intercept	-2.76	0.35	0.004
	Treatment	0.47	0.32	0.14
	Parity, 1 vs >1	-0.33	0.17	0.05
	Calving month			<0.001
	Calving month x Treatment			0.09
Metritis	Intercept	-1.99	0.16	0.001
	Treatment	0.10	0.12	0.40
	Parity, 1 vs >1	0.52	0.12	<0.001
	Retained placenta	1.93	0.16	<0.001
Endometritis	Intercept	-4.11	0.34	0.001
	Treatment	0.27	0.28	0.34
	Parity, 1 vs >1	0.03	0.23	0.90
	Calving month			0.01
	Retained placenta	0.99	0.27	<0.001
	Metritis	1.53	0.31	<0.001
	Metritis x Treatment	-0.90	0.44	0.04

Reference groups: Control group, month 3, no RP (retained placenta); no Metritis.

Parity was associated with the occurrence of RP, as multiparous cows showed a higher occurrence than primiparous cows (*P* = 0.05). Calving month showed a significant association (P < 0.001) and the calving month by treatment interaction tended to significance (P = 0.09). No overall treatment effect (type III test) was detected (Control = 8.1%, PEG = 8.9%; P = 0.54).

For metritis, there was no significant treatment effect (*P* = 0.40), while parity was associated with its incidence (*P <* 0.001); primiparous cows showed a higher incidence than multiparous cows. There was no treatment by parity interaction. No overall treatment effect (type III test) was detected (Control = 32.2%, PEG = 34.5%; P = 0.37).

**Fig 4:**
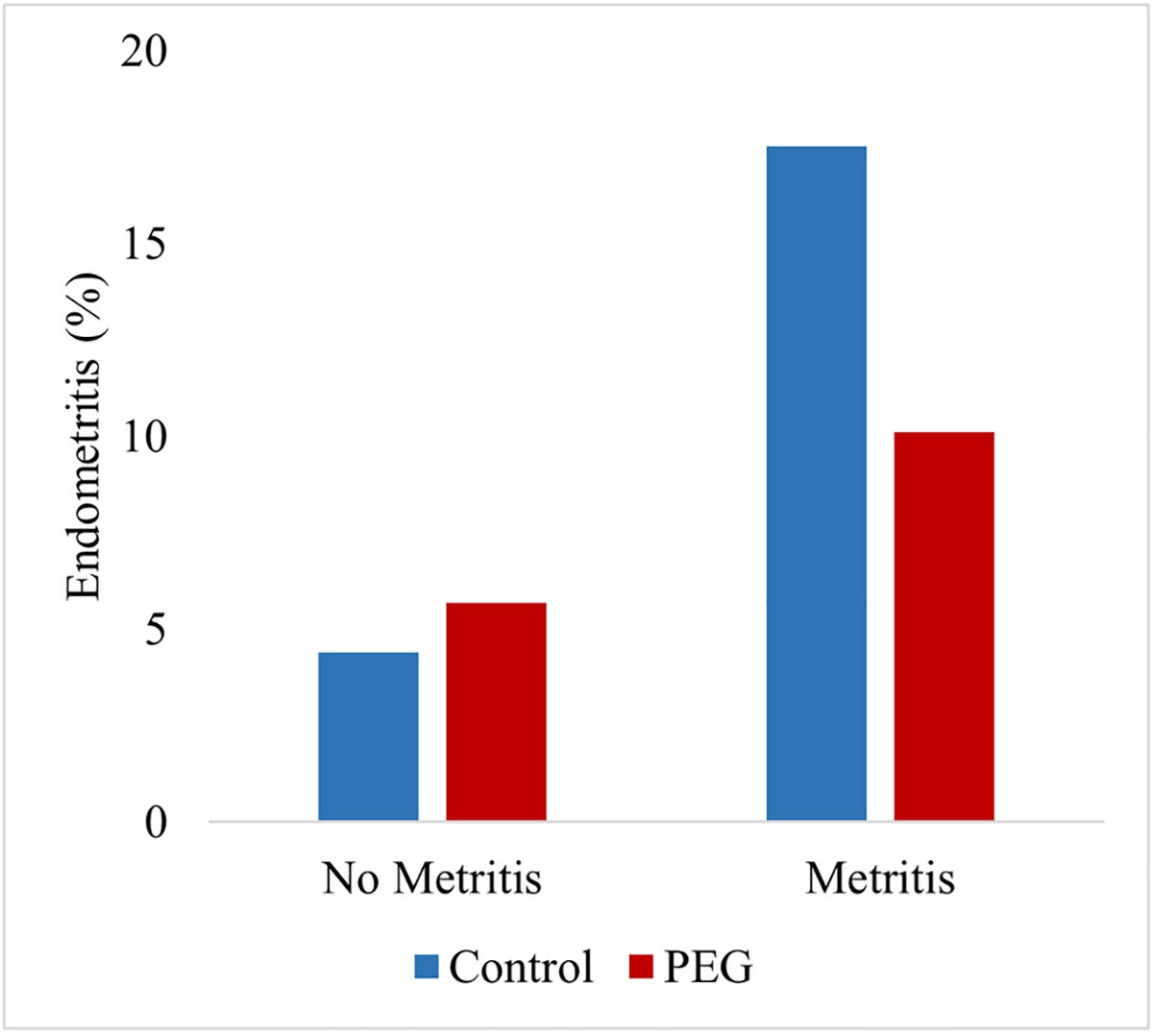
Cows with a diagnosis of endometritis (%) by previous metritis diagnosis. No metritis: Control = 837, PEG = 778; Metritis: Control = 177, PEG = 177. There was a significant interaction between treatment and the previous occurrence of metritis *P* = 0.04.

For endometritis, model results showed no treatment (*P* = 0.34) or parity effects (*P* = 0.90). There was no treatment by parity interaction. Occurrence of RP (*P* < 0.001) and metritis (P < 0.001) increased the risk for endometritis. A significant interaction of treatment with the previous occurrence of metritis was detected (*P* = 0.04). Fig 4 presents endometritis occurrence in cows with and without a previous metritis case in Control and PEG cows. In cows with metritis, PEG treatment reduced the occurrence of subsequent endometritis by 42.3% (Control = 17.5%, PEG = 10.1%). No overall treatment effect (type III test) was detected (Control = 9.0%, PEG = 7.6%; *P* = 0.40).

## Discussion

In this randomized controlled trial on four commercial grazing dairy farms, we investigated whether PEG treatment affected CM and uterine disease occurrence during a full lactation in primiparous and multiparous grazing dairy cows. We also evaluated the association of Pre-BCS and Pre-NEFA concentration and relevant interactions with disease occurrence. Treatment with PEG reduced the occurrence of a first case of CM during the first 30 DIM in grazing dairy cows and reduced the hazard of a first case and the rate of total cases of CM during the full lactation. The treatment effects were independent of parity. Also, for the first time, we showed that Pre-BCS interacts with PEG; in over-conditioned cows, PEG reduced the occurrence of a first case of CM by almost 50% during the first 30 DIM. The hazard analysis of a first case of CM during a full lactation suggested that the preventive effect of PEG disappeared with increasing DIM. Finally, PEG treated cows with metritis subsequently showed a reduced occurrence of endometritis compared to control cows with metritis.

Treatment with PEG resulted in an overall reduction of almost 25% in the occurrence of a first case of CM during the first 30 DIM. The beneficial effect of PEG decreased with increasing DIM (Table 3, Fig 3). Even so, PEG treated cows had fewer total cases of CM per day at risk (Table 4). To our knowledge, this study is the first that follows the treated animals during a full lactation. The preventive effect of PEG on CM during the first 30 DIM is in contrast with Zinicola et al. [14], whose study included only cows with an acceptable BCS. Our results are compatible with others that included cows regardless of their BCS, in which a similar effect of PEG on CM was reported, for example the 34% reduction reported by Canning et al. [12] and the 25% reduction reported by Ruiz et al. [13]. This transient beneficial effect of PEG is biologically plausible since immunosuppression is typically highest during early lactation [2, 9] and the effect of PEG on WBC counts is transient [32]. The postpartum beneficial effect is especially valuable since early lactation CM cases can be more severe compared with mid or late lactation cases [33]. Also, early lactation CM increases the risk of subsequent CM cases [34]. The reduction in early lactation CM could be a possible explanation for the preventive effect of PEG on the total cases of CM during the full lactation. It could also be hypothesized that during early lactation PEG increases the cure rate after antibiotic treatment. Future analyses on these data will be done to evaluate this. Indeed, the ability to recruit neutrophils into the mammary gland is essential for mastitis resolution [35], and Powell et al. [36] reported that PEG activated neutrophils for quick recruitment to the infected mammary gland, reduced severity of CM and lowered bacterial shedding in milk, suggesting an improved mastitis resolution. Early lactation CM impairs reproductive performance and increases the culling rate [34, 37]. Whether the preventive effect of PEG on the occurrence of CM during early lactation reported here could affect the reproductive performance and culling warrants further analysis of our data.

Our results showed that over-conditioned control cows had a higher occurrence of first cases of CM during the first 30 DIM and total cases of CM compared to cows with an acceptable BCS (Fig 2, Table 4). It has been reported before that cows with a high BCS had more mastitis relative to cows with a moderate BCS [7], and these authors suggest that this is related to an impaired energy and lipid metabolism that may affect the immune response. Over-conditioned cows mobilize more reserves than cows in an acceptable body condition [7], leading to increased postpartum concentrations of NEFA [38] and beta-hydroxybutyrate [7], both metabolites being associated with an impaired immune response during early lactation [2]. Interestingly, we found a Pre-BCS by treatment interaction, as PEG reduced the first cases of CM by almost 50% in over-conditioned animals, while no effect was observed in under-conditioned cows or cows with an acceptable BCS (Fig 2). These data could explain the lack of treatment effect found in the study reported by Zinicola et al. [14], as they excluded low and high BCS cows (BCS < 3 and > 3.75).

The Pre-NEFA by treatment interaction did not reach statistical significance (*P* = 0.15; Table 2); however, the inclusion of this interaction in the final model changed the treatment estimate considerably. This suggests that PEG has a differential effect according to the Pre-NEFA category. We expected a stronger association based on our recent findings that PEG reverts the negative association between Pre-NEFA and neutrophil counts [11]. Analysis of the estimated least squares means differences by Pre-NEFA category shows that, in high Pre-NEFA cows, PEG treatment significantly reduced the occurrence of a first case of CM during the first 30 d of lactation by 35%. Melendez et al. [4] used a very high cut-off (1.2 m*M*) of NEFA at calving to show an association with CM; however, as far as we know, no cut-off for Pre-NEFA was described as a risk factor for CM. Based on the literature [6] we performed our analyses using 0.3, 0.4 and 0.5 as cut-off points, and using a cut-off of 0.5 resulted in the smallest probability of committing a type I error. As mentioned before, the effect of PEG was strongly dependent on Pre-BCS, and Pre-BCS has been associated with peripartum NEFA concentrations [7, 39, 40], which may partially explain our results. Overall, the beneficial effect of PEG treatment on the occurrence of a first case of CM during the first 30 DIM in over-conditioned cows suggests that PEG treatment reduces the potential negative effect of metabolic stress on the immune system, to demonstrate it, further studies measuring immune system markers are warranted.

Pegbovigrastim showed no effect on RP incidence. These results are in accordance with those reported by Zinicola et al. [14]. On the other hand, Ruiz et al. [13] reported a reduction in the incidence of RP in PEG treated cows. A preventive effect of PEG on RP occurrence could be expected, since diminished neutrophil functional capacity is mentioned in the etiology of the disease [41], and PEG improves neutrophil functional capacity [9, 10, 42]. Our data show that treatment interacted with calving month; nonetheless, we cannot make inferences about this finding since the present study was not designed to test the calving month effect and thus, further research will be needed to identify factors that could explain this relationship.

Metritis occurrence was not affected by treatment, which is in accordance with a previous report [14]. Ruiz et al. [13] reported a higher incidence of metritis in PEG treated cows. The study of Zinicola et al. [14] and the present work were especially designed to assess all cows by trained technicians at the same time point (one week after calving approximately), while in the study of Ruiz et al. [13] metritis diagnoses relied on trained field workers but without formal assessment protocols. A more robust vaginal discharge due to a greater intra-uterine neutrophil influx in PEG treated cows was suggested by Ruiz et al. [13]. This hypothesis is supported by the findings of lower blood neutrophil counts [11] and higher neutrophil counts in the vagina in PEG cows with metritis compared to PEG cows without metritis [14]. As expected [22], primiparous cows had a higher metritis occurrence, while no treatment by parity interaction was detected. A study using primiparous cows on one German farm reported that treatment with PEG reduced the occurrence of acute puerperal metritis [15], but, due to the small sample size and the high incidence of the disease, these conclusions should be evaluated with caution.

Interestingly, treatment with PEG reduced (42.3%) endometritis occurrence in animals that were diagnosed with metritis previously (Fig 4). High proportions of neutrophils in the endometrium soon after calving were reported as beneficial to uterine health and subsequent fertility [43]. These authors reported that cows that are capable of recruiting large numbers of neutrophils rapidly to the uterus in the immediate postpartum period are less likely to suffer bacterial infections and more likely to have a healthy postpartum uterine involution. The lower endometritis occurrence in PEG treated cows with metritis would suggest that a higher neutrophil influx to the uterus due to PEG treatment improves the healing process. Endometritis on its own or as a consequence of metritis has a strong deleterious effect on reproductive performance and increases the chance to be culled for reproductive failure compared to cows without endometritis [22, 44]. Whether our findings affect the reproductive performance and the risk of culling warrants further analysis.

## Conclusions

Overall, treatment with PEG reduced the occurrence of a first case of CM during the first 30 DIM in grazing dairy cows and reduced the hazard of a first case and the rate of total cases of CM during the full lactation. We showed that these effects were independent of parity. Also, for the first time, we showed that Pre-BCS interacted with PEG; in over-conditioned cows, PEG strongly reduced the occurrence of a first case of CM during the first 30 DIM. The hazard analysis of first cases of CM during the full lactation suggested that the preventive effect of PEG disappeared with increasing DIM. Pegbovigrastim treated cows with metritis subsequently showed a reduced occurrence of endometritis compared to control cows with metritis. Pegbovigrastim reduces the occurrence of CM particularly in cows at risk of elevated lipid mobilization, and PEG ameliorates the uterine healing process in cows that experienced metritis.
